# HIV-1 subtypes and drug resistance mutations among female sex workers varied in different cities and regions of the Democratic Republic of Congo

**DOI:** 10.1371/journal.pone.0228670

**Published:** 2020-02-11

**Authors:** Eun Hee Kwon, Godefroid M. A. Musema, Jessica Boelter, Sydney Townsend, Désiré Tshala-Katumbay, Patrick K. Kayembe, John West, Charles Wood

**Affiliations:** 1 Nebraska Center for Virology, University of Nebraska-Lincoln, Lincoln, Nebraska, United States of America; 2 School of Biological Sciences, University of Nebraska-Lincoln, Lincoln, Nebraska, United States of America; 3 Department of Biochemistry, University of Nebraska-Lincoln, Lincoln, Nebraska, United States of America; 4 School of Public Health, University of Kinshasa, Kinshasa, Democratic Republic of Congo; 5 Department of Neurology, School of Medicine and School of Public Health, Oregon Health & Science University, Portland, Oregon, United States of America; 6 Department of Neurology, University of Kinshasa, Kinshasa, Democratic Republic of Congo; 7 Institut National de Recherches Biomédicales, Kinshasa, Democratic Republic of Congo; University of Cincinnati College of Medicine, UNITED STATES

## Abstract

**Background:**

Complex mosaic structures of HIV-1 were found in the Democratic Republic of Congo (DRC). Currently, there is limited information on the circulating HIV-1 strains, the distribution of these strains and antiretroviral (ART) resistant viruses in different regions of the country, and the HIV-1 strains harbored by the high-risk groups like female sex workers (FSW) reported to be the source of recombinant and ART resistant viruses.

**Methods:**

Dried Blood Spots (DBS), collected from 325 infected FSWs in ten cities from 2012 DRC HIV/STI Integrated Biological and Behavioral Surveillance Survey, were tested for HIV-1 genotypes and antiretroviral resistance mutations. Regional segregation of HIV-1 clades was detected using phylogenetics. The significance for differences in HIV-1 subtype and drug resistance mutations were evaluated using Chi-square tests.

**Results:**

There were 145 (*env*) and 93 (*pol*) sequences analyzed. Based on *env* sequences, the predominant subtype was A1 (44%), and recombinants as defined *pol* sequences comprised 35% of the total sample. Paired sequences of *pol* and *env* from DRC FSW revealed mosaic recombinant in 54% of the sequences. Distinct geographic distributions of different HIV-1 subtypes and recombinants were observed. Subtype A1 was prevalent (40%) in Goma located in the East and significantly higher than in Mbuji-Mayi (*p*<0.05) in the South-central region, or in Lubumbashi in the South. Antiretroviral resistance was detected in 21.5% of 93 *pol* sequences analyzed, with the M184I/V and K103N mutations that confer high-level resistance to NRTI and NNRTI, respectively, being the most frequent mutations. However, the K103N mutant viruses were found only in the East.

**Conclusion:**

HIV-1 variants found in DRC FSW reflect those reported to circulate in the general population from the corresponding geographical locations. HIV-1 mosaic genetics were readily detected in FSW. Importantly, ART resistance mutations to NNRTI and NRTI were common in the DRC sex workers.

## Introduction

The group M Human Immunodeficiency Virus Type-1 (HIV-1) epidemic likely originated from Kinshasa in the Democratic Republic of Congo (DRC), and subsequently disseminated throughout the country via rail and river traffic in the 1960s, before spreading globally[1–3]. Rapid global dissemination of HIV-1 M subtypes resulted in evolution of nine distinct subtypes (A-D, F-H, J and K), and more than 96 circulating recombinant forms (CRF), and other unique recombinant forms (URF) (https://www.hiv.lanl.gov/content/sequence/HIV/CRFs/CRFs.html). The number of HIV-1 recombinants has increased temporally to a current estimate of >20% of global infections[4]. The increase in recombinants suggests that such genotypes will continue to play a major role in the HIV epidemic. In West and Central African countries a variety of HIV-1 subtypes and recombinant forms continue to co-circulate and unique infectious HIV-1 recombinant forms are frequently detected [5].

As one of the first countries affected by the AIDS epidemic, DRC, has an epidemic characterized by a complex population of HIV-1 subtypes and divergent HIV recombinant variants. Due to recombination, high frequency discordance in subtype assignments between *pol* and *env* (41.1–59.3%) in DRC samples has been reported [6, 7]. Similar HIV-1 diversity and divergence is now increasingly evident in other countries[1, 8]. The frequency of recombinant HIV-1 forms in DRC could derive from sequential or concurrent infections of individuals with more than one strain of HIV-1[9]. Intermolecular recombination between two distinct HIV-1 genomes requires co-infection of single cells resulting in strand cross-overs during reverse transcription. While recombination events resulting in replication competent and adequately fit progeny are predicted to be low probability events, such probabilities are likely enhanced in high risk populations, such as the female sex workers (FSWs) due to high rates of superinfection[10–12]. Hence, understanding HIV-1 transmission and subtype genetic diversity in high risk DRC populations is likely to provide insight into pathways of viral evolution and response to therapy.

In fact, according to the 2012 DRC HIV/STI Integrated Biological and Behavioral Surveillance Survey (IBBS), the HIV prevalence in female sex workers was 6.97% (414/5953), compared to a >4-fold lower HIV-1 prevalence among females between age 15–49 in the general population[13, 14]. While it is logical that FSWs are more likely to harbor multiple HIV-1 variants and recombinants, the prevalence of subtypes and of CRFs in this population has not been documented. In addition, since HIV-1 subtypes tend to segregate geographically based on fitness and founder effects, the geographic distribution of HIV-1 subtypes and recombinants needs to be determined.

Since drug resistant viruses reduce the efficacy of HIV treatment and result in virological failure (VF), the presence of ART resistant genotypes is a concern. Since FSWs are often expose to unprotected sexual contact with their clients, and have higher probability of infection, or infection with multiple HIV-1 genotypes, therefore we anticipated that FSW might also harbor variants with drug resistance mutations (DRMs) at high prevalence. The most common first-line ART regimen used in the DRC is a nucleoside reverse transcriptase inhibitor (NRTI)/non-nucleoside reverse transcriptase inhibitor (NNRTI) combination of stavudine (d4T)/azidothymidine (AZT), lamivudine (3TC) and nevirapine (NVP)/efavirenz (EFV). For the second-line therapy, a combination of abacavir (ABC), didanosine (ddI) and ritonavir boosted lopinavir (LPV/r) is used [15, 16]. ART has reduced AIDS- related deaths, but parallel with the ART scale-up, an increase of VF[17] and emergence of mutations coupled with VF was observed in the DRC[15, 16]. Importantly, ART scale-up coincided with increases in detection of DRMs in treatment naïve patients, from 7.9% in 2008 to 10% in 2013–2014. Such increases included a high proportion of DRMs that limit the effectiveness of the first-line ART regimen utilized in the DRC[15, 18].

Despite the potential of FSWs to be carriers and transmitters of CRFs, and drug resistant HIV-1 strains in DRC, limited investigation has been conducted in this population. Using dried blood spots (DBSs) collected from HIV-1 positive FSWs sampled in the 2012 DRC IBBS we investigated co-circulating HIV-1 genotypic variation and DRMs. Our study is the first to demonstrate distinct genotypic segregation of HIV-1 and DRMs among FSWs from different geographical locations of the DRC. These individuals and their clients represent potential sources of transmission to uninfected and treatment naïve individuals, leading to the generation of recombinants and variants that may increase rates of ART failure in the near future.

## Materials and methods

### Sample collection

As part of the 2012 IBBS, 5,953 FSWs aged 15 to 49 participated in HIV-1/STI surveillance. The original study protocol was reviewed and approved by the Institutional Review Boards (IRBs) of Kinshasa School of Public Health, Democratic Republic of the Congo and the Division of Global HIV/AIDS (DGHA) at Center for Disease Control and Prevention (CDC). Study participation was strictly voluntary and measures were taken to ensure the respect, dignity and freedom of participants. Complete participant confidentiality was guaranteed. After explaining study purpose, written informed consent was obtained from each respondent for blood sampling. Study participants under age 18 were considered as emancipated minors who are responsible for their lives including foods, clothes, and housing. Thus, parental consent was not required for them. All biological specimens were labeled with a study number unlinked to the participant’s name or other identifiers. Only deidentified blood spot specimens were analyzed by study personnel at University of Nebraska-Lincoln. FSWs were randomly selected from their working sites, including bar and dance halls in 11 cities: Bukavu (Sud Kivu-province), Goma (Nord Kivu-province), Kananga (Kasai-Occidental-province), Kikwit (Bandundu-province), Kindu (Maniema-province), Kinshasa (Kinshasa-province), Kisangani (Orientale-province), Lubumbashi (Katanga-province), Matadi (Bas-Congo-province), Mbandaka (Equateur-province) and Mbuji-Mayi (Kasai-Oriental-province). Blood specimens were collected between December 2012 and January 2013. HIV infection was confirmed by the DRC HIV rapid test algorithms, followed by enzyme-linked immunosorbent assay (ELISA) to confirm the positive results.

### DNA extraction and amplification of HIV *env* and *pol* sequences

We obtained properly preserved 325 Dried Blood Spots (DBS) from HIV positive FSWs from ten DRC cities. DBSs from Kikwit were improperly preserved, and were excluded. Genomic DNA was isolated from DBS using Qiagen lysis buffer with proteinase K as described previously[19]. To remove any remaining protein or lipid, extracted nucleic acids were subjected to phenol-chloroform biphasic extraction. To verify DNA extraction and its capacity to serve as a template for polymerase chain reaction (PCR) amplification, 50ng of extracted DNA was subjected to PCR using primers for the human beta-actin gene: ACTIN1 [5’-TTCTACAATGAGCTGCGTGT-3’] and ACTIN2 [5’- GCCAGACAGCACTGTGTTGG-3’] and evaluated by TAE-agarose gel electrophoresis. Only those samples producing a detectable 636bp amplification product for human beta-actin were subjected to amplification for HIV-1 proviral sequences to ensure the quality of the DNA obtained. All DBS amplifications were controlled with a no-template control reaction as well as a positive specimen with amplifiable HIV positive human genomic DNA (8E5 cells).

For subtyping, two regions of the HIV-1 genome, 1493bp of *pol* encoding the protease and reverse transcriptase (PI-RT): outer primers [5’-GCAAGAGTTTTGGCTGAAGCAATGAG-3’] and [5’- CCTTGCCCCTGCTTCTGTATTTCTGC-3’]; inner primers [5’- TGCAGGGCCCCTAGGAAAAAGGGCTG-3’] and [5’- CATGTACCGGTTCTTTTAGAATCTCTCTGTT-3’], and 593bp of *env* (c2-v4): outer primers [5’- CCAATTCCCATACATTATTGTGCCCCAGCTGG-3’] and [5’- CCAATTGTCCCTCATATCTCCTCCTCCAGG-3’]; inner primers [5’- GTCAGCACAGTACAATGACACATGG-3’] and [5’- TCCTTGGATGGGAGGGGCATACATTGC-3’] were amplified by nested PCR using Takara Ex Taq DNA polymerase (Clontech, Takara Bio USA, Mountain View, CA)[6, 20]. The amplified fragments were purified using E.Z.N.A.® Gel Extraction Kit (Omega, GA, USA). Alternative *env* primers were employed for samples that were not amplifiable by the *env* (c2-v4) primers [21]. The purified PCR products were sequenced using BigDye^TM^ Terminator v3.1 Cycle Sequencing Kit (Applied Biosystems) with partially overlapping sequencing primers, and the product was analyzed with Eurofins MWG Operon.

### HIV-1 subtype assignment

To trim and generate the contigs from the *pol* and *env* sequences we used Bioedit v7.2.5. Contigs from each participant were aligned to the HXB2 (GenBank Accession No. K03455.1), 2133–3445 nt for *pol* and 7011–7496 nt for *env*. The Stanford REGA HIV-1 Subtyping Tool v.3.0 was utilized to identify the HIV-1 subtype for both *pol* and *env* sequences[22]. However, some *env* sequences could not be assigned to a subtype by REGA due to lack of high bootstrap value phylogenetic identity with any acceptable REGA reference sequence. For these sequences, the jumping profile Hidden Markov Model (jpHMM) was utilized to assign HIV-1 subtypes or recombinant forms[23]. In addition, the *pol* genes assigned to recombination by REGA were further analyzed for a confirmation of recombination and identify possible recombinant breakpoint locations using Simplot version 3.5.1[24]. The parameters of bootscan analysis in the Simplot were as follows: Neighbor-Joining using the Kimura 2-parameter model, 100 bootstrap replication, window and step size of 400bp and 40bp, respectively, gap-strip: on, and T/t: 2.0. The reference sequences of HIV-1 group M *pol* subtype including recombinants used for Simplot were obtained from the HIV sequence database of the Los Alamos National Laboratory (https://www.hiv.lanl.gov/content/sequence/NEWALIGN/align.html). Sequences obtained from FSWs were deposited in GenBank under accession numbers MK133142-MK133234.

### Phylogenetic tree analysis

Phylogenetic trees were constructed from the alignment of 93 HIV-1 *pol* DNA sequences and HIV-1 group M *pol* subtype/CRF reference sequences obtained from HIV sequence database to investigate any regional segregation of specific HIV-1 subtypes. The sequence alignment was established using CLUSTAL W[25]. Then the trees were constructed from the aligned sequences based on the maximum likelihood method (Tamura-Nei model) with 1000 bootstrap resampling within the MEGA 7 program[26, 27]. Recombinants that did not cluster with the pure subtype and CRF reference sequences were run through BLAST against the HIV database (https://www.hiv.lanl.gov/content/sequence/BASIC_BLAST/basic_blast.html) to identify similar sequences from previous studies. Related sequences were then added to the reference sequences (accession no. AM041051, MF372645).

### Detection of drug resistance mutations

Sequences from the *pol* gene were submitted to the Stanford HIV Drug Resistance Database to detect drug resistance mutations and their potency scores.

### Statistical analysis

Overall and regional prevalence of HIV, HIV-1 subtypes, and drug mutations were calculated in FSWs. The prevalence means and standard deviation (S.D.) of HIV-1 subtypes between the cities with highest HIV-1 prevalence were evaluated as indicated in Table 1. Differences in prevalence of subtypes and DRM between the DRC regions were tested using Chi-square and *p* values <0.05 were considered statistically significant.

**Table 1:** Varied proportion of HIV-1 subtypes in the four cities with highest HIV-1 prevalence by comparison the classified subtypes obtained from paired *pol* and *env*.

Cities	A1	C	D	G	Recombinants	Total sample
Mbuji-Mayi	3 (13.0%)	5 (21.7%)	1 (4.3%)	1 (4.3%)	13 (56.5%)	23
Lubumbashi	0	3 (30.0%)	1 (10.0%)	0	6 (60.0%)	10
Kinshasa	3 (23.0%)	1 (7.7%)	0	1 (7.7%)	8 (61.5%)	13
Goma	10 (40.0%)	6 (24.0%)	0	0	9 (36.0%)	25
Percentage Mean	19.0%	20.85%	3.58%	3.0%	53.5%	99.9%
S.D.	16.87	9.44	4.74	3.73	11.85	NA

## Results

### HIV-1 prevalence in female sex workers

**Fig 1:**
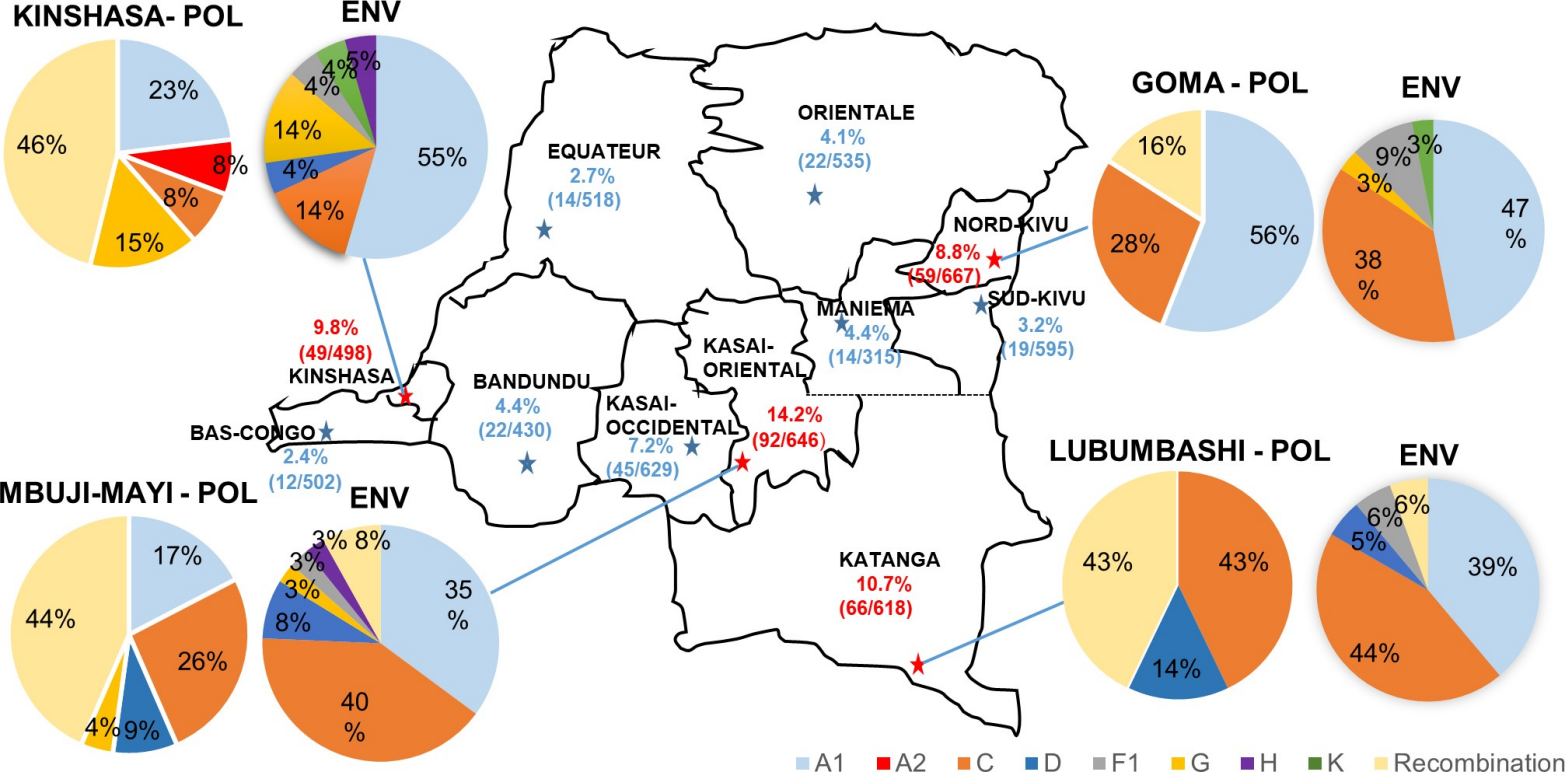
HIV-1 prevalence and regional distribution of HIV-1 variants detected in the DRC. The four cities with highest prevalence of HIV infection are indicated by red stars ★. Pie charts represent the proportion of each HIV-1 subtype as determined by *pol* and *env* gene sequences in these four locations, and the different clades were color coded as shown in the figure. Prevalence in other cities is indicated by blue stars ★.

The IBBS (2012) revealed heterogeneous geographic distribution of HIV-1 infections in FSWs across the country. In terms of HIV-1 prevalence by city, as determined by HIV rapid diagnosis test, Mbuji-Mayi located in Kasai-Oriental province (14.2%) ranked highest, followed by Lubumbashi in in Katanga (10.7%), Kinshasa in Kinshasa (9.8%), Goma in Nord-Kivu (8.8%), Kananga in Kasai-Occidental (7.2%), Kindu in Bandundu (4.4%), Kisangani in Orientale (4.1%), Bukavu in Sud-Kivu (3.2%), Mbandaka in Equateur (2.7%), and Matadi in Bas Congo (2.4%)[28] (Fig 1). Interestingly, the four cities with highest HIV-1 prevalence are located in the different regions of DRC, Mbuji-Mayi is located in the south-central region, Lubumbashi in the South, Kinshasa in the West and Goma in the East.

### HIV-1 genetic diversity in infected FSWs

Among 325 DBSs analyzed, 63% (205 cases) produced amplifiable sequences. E*nv* gene sequences were obtained from 145 individuals, which were subsequently used for clade and recombinant designations. The rest were either unreadable or contained multiple overlapping sequences, and were excluded from the analyses. Of those 145, we then further sequenced the *pol* sequences to determine potential recombinants. Out of the 145 amplifiable samples, we obtained 93 *pol* sequences for determination of recombination or subtype discordance between the paired *env* and *pol* genes. The remaining specimens did not PCR amplify *pol* due either to low HIV-1 copies, or inadequate primer annealing resulting from sequence variation in the primer binding sites[18]. The 93 individual *pol* and 145 *env* sequences analyzed segregated geographically as follows: 23 (*pol)* and 37 (*env*) from Mbuji-Mayi, 10 (*pol)* and 18 (*env*) from Lubumbashi,13 (*pol) and* 22 (*env*) sequences from Kinshasa, 25 (*pol)* and 32 (*env*) from Goma, , 4 (*pol)* and 8 (*env*) from Kananga, 2 (*pol)* and 5 (*env*) from Kindu, 4 (*pol)* and 5 (*env*) from Kisangani, 6 (*pol)* and 9 (*env*) from Bukavu, 3 (*pol)* and 5 (*env*) from Mbandaka, and 3 (*pol)* and 4 (*env*) from Matadi (S1 Table).

**Fig 2:**
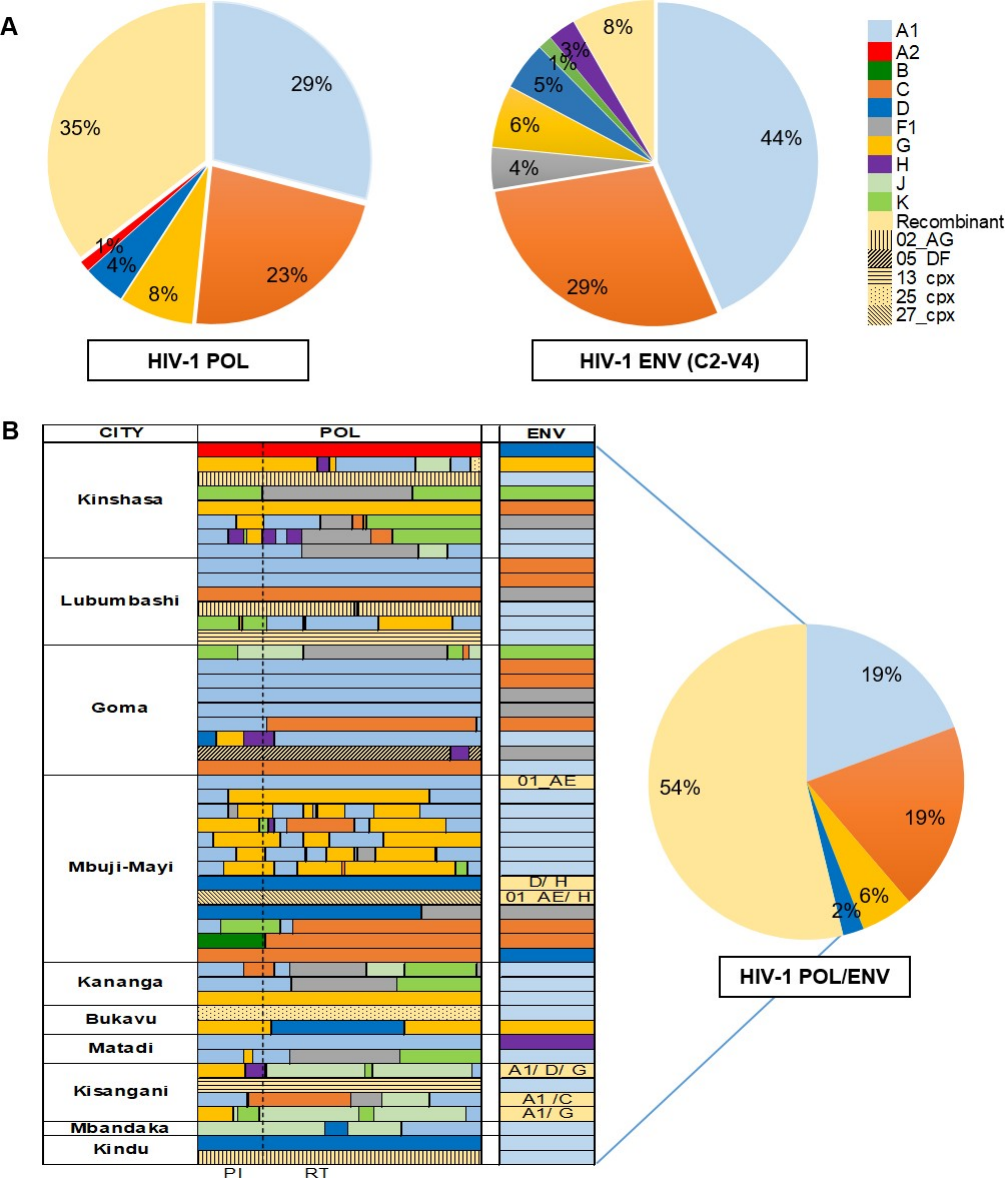
The overall HIV-1 genetic diversity and circulating recombinants observed in ten cities. (A) The proportion of HIV-1 subtypes based on the *pol* and *env* sequences, respectively. The different clades are color coded as shown. (B) Representation of the mosaic structure in the *pol* genes (HXB2 2133–3445 nt) by Simplot without bootstrap threshold. The approximate breakpoints between subtypes were analyzed by bootscanning analysis of *pol* genes with the following conditions: window: 400bp; step: 40bp; gap-strip: on; T/t: 2.0; Neighbor-Joining; Kimura (2-parameter); 100 bootstrap replication. The proportion of HIV-1 subtypes in each of the ten cities based on the paired sequences of *pol* and *env*. The different clades are color coded as shown and the types of recombinant as determined by the *pol* and *env* sequences are as indicated. Structures of recombinants were determined by parallel comparison of HIV-1 strains between *pol* and *env*. For subtyping, REGA V3 subtyping tool and jpHMM were utilized.

HIV-1 group M subtypes and recombinants co-circulating among FSWs varied in different cities, and their prevalence, based on the *env* and *pol* gene sequences detected, is shown in Figs 1 and 2A. Among the ten DRC cities analyzed, the *env* sequence subtype hierarchy was A1 (44%), followed by C (29%), recombinant (8%), G (6%), D (5%), F1 (4%), H (3%) and K (1%). However, the dominant form, based on the *pol* sequences, was recombinant (35%), followed by A1 (29%), C (23%), G (8%), D (4%), and A2 (1%). The recombinant structures in the *pol* genes analyzed by Simplot were shown in Fig 2B. When the *pol* and *env* sequences were combined for subtype analysis, 54% (50/93) exhibited subtype discordance between the two genes (Fig 2B), likely as a result of recombination.

**Fig 3:**
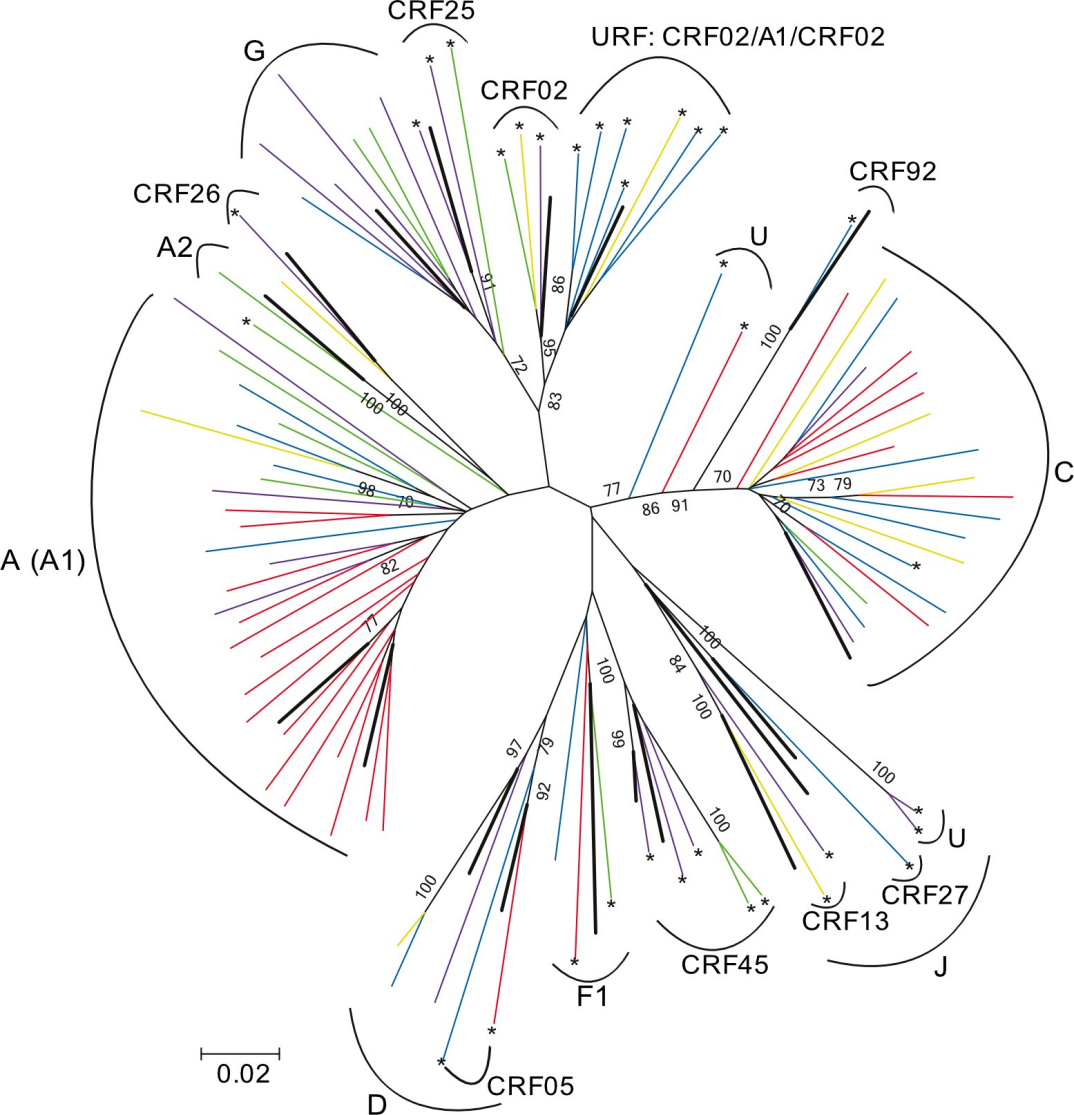
Maximum-likelihood phylogenetic analysis of aligned nucleotide sequences on the HIV-1 *pol* (HXB2 2133–3445 nt). Different geographic locations are color-coded as follows: blue, Mbuji-Mayi; yellow, Lubumbashi; green, Kinshasa; red, Goma; purple, Kananga, Bukavu, Mbandaka, Kindu, Matadi, and Kisangan. Sequences were aligned with HIV-1 M subtypes and recombinants reference sequences. Reference strains are represented by thicker lines, and the recombinants identified using the REGA V3 subtyping tool are indicated with an asterisk (*). Only bootstrap values >70 are indicated above the branches.

Utilizing subtyping tools and phylogenetic analyses, the CRFs in *pol* were shown to include CRF02_AG, CRF05_DF, CRF13_cpx, CRF 25_cpx, CRF26_A5U, CRF27_cpx, CRF45_cpx, and CRF92_C2U (Fig 3). The distribution of CRFs varied by location. Among them, CRF45 (5/93, 5.4%) was the most prevalent, but interestingly it was present only in the western region of DRC: Kinshasa, Matadi, and Kananga. Geographic patterning for a sister group of CRF02 frequently detected as a URF (7/93, 7.5%). This genotype was defined by BLAST search as CRF02/A/CRF02 (accession number AM041051) [7]. These URFs were more commonly detected in the South-central part of DRC: 6 from Mbuji-Mayi and 1 from Lubumbashi. Since the other CRFs and URFs were only found sporadically in individuals from different regions, we were not able to investigate geographical relationships. We detected recombinants in cities with lower HIV-1 prevalence in FSWs; however, due to insufficient sampling at those sites, we were not able to determine whether those recombinants were predominant in those locations.

### Distribution of HIV-1 variants in cities with highest HIV-1 prevalence

Based on the discrete geographic pattern of recombinants demonstrated above, we then evaluated the geographic distribution of HIV-1 *pol* and *env* sequence variants circulating in four cities with the highest HIV prevalence: Kinshasa, Mbuji-Mayi, Lubumbashi (a mining town where HIV Group M is thought to have arrived in the 1930s from Kinshasa)[3], and Goma (where violent conflict has been taking place since 1996) (Fig 1). The most frequent subtype discordance, between *pol* and *env* occurred in Kinshasa (8/13, 61.5%), followed by Lubumbashi (6/10, 60.0%), Mbuji-Mayi (13/23, 56.5%), and Goma (9/25, 36.0%) (Table 1). Interestingly, Goma was the place with the highest prevalence of HIV subtype A1 (40%) (Table 1), and the differences in subtype A1 prevalence between Goma and Mbuji-Mayi (*p* = 0.0374), and Goma and Lubumbashi (*p* = 0.0197) were significant (*p* <0.05). Phylogenetics confirmed that the subtype A1 strains from Goma clustered together (Fig 3). In addition, a trend toward higher prevalence of subtype C was detected in the south-central region (Mbuji-Mayi), East (Goma), and South (Lubumbashi), whereas Kinshasa had low subtype C prevalence. The differential subtype C prevalence by city was however not statistically significant at *p*>0.05 due to insufficient sampling in outlying areas.

### Drug resistance mutations and their distributions among FSWs in different cities

Given the potential risk in transmission of drug resistance viruses to the general population from this group, we then quantified the presence of DRMs in the HIV-1 *Pro* and *RT* sequences we obtained. Natural polymorphisms between subtypes were not included in the analyses. We identified 20 cases (21.5%) that harbored DRMs among the 93 *pol* amplifiable DBSs, and a number of them were found to have FTC/3TC and NVP/EFV resistance, consistent with the first-line ART regimens used in the DRC (Table 2). Among the 20 cases identified, the most common mutations were against NNRTIs. Ten FSWs were found to harbor only NNRTI resistant mutations, 3 were found to have only NRTI resistance, and 3 only PI resistance. The remaining 4 cases were found to harbor both NNRTI and NRTI resistance mutations. With the 14 individuals who had NNRTI-resistance mutations, two had multiple NNRTI mutations. The K103N mutation was the most common, followed by E138A/G, G190A and A98G, and then V179D/E/T and Y181C (Table 2). The K103N, Y181C, and G190A are known to confer high-level resistance to NVP, and intermediate to high resistance to EFV. Among the 7 cases with NRTI mutations, the most common was M184I/V, which is known to confer high-level resistance to both emtricitabine (FTC) and 3TC. Other NRTI resistance mutations were K65R and K219R. The two non-polymorphic accessory mutations associated with PI mutations were L33F and I54V, and were observed in three PI resistant patients. The mutation scores, which represent the levels of resistance to anti-retroviral are shown in Table 2.

**Table 2:** Summary of HIV-1 drug resistance mutations observed in 20 female sex workers in the DRC.

Sequence includes PR: codons 1 - 99						
Sequence includes RT: codons 1 - 298						
PROVINCE	DR Prevalence	ID	HIV CLADE	HIV DRUG MUTATIONS (SCORES)		
			POL	PI	NRTI	NNRTI
Mbuji-Mayi in Kasai-Oriental	4/23 (17.4%)	2-H1	C		M184I (115)	
		3-E6	B/C			E138A (25)
		3-D4	A1/K/A1/C			V179T (0)
		3-A3	D		K219R (20)	
Lubumbashi in Katanga	2/10 (20%)	4-B7	D			E138G (45)
		1-B7	A	I54V (30)		
Kinshasa in Kinshasa	2/13 (15.4%)	1-D9	K/F1/K			E138A (25)
		1-E8	A1/G/A1/F1/C/G/K		M184V(115)	G190A (130)
Goma in Nord-Kivu	8/25 (32%)	2-A6	A			K103N (120)
		2-A7	C		K65R (150)	
		2-C3	A	L33F (15)		
		2-D4	A1/C	L33F (15)		
		2-E2	CRF05_DF			K103N (120)
		2-E8	A		M184V (115)	A98G, K103N (190)
		2-D8	D/G/H/A1			K103N (120)
		2-C6	C			E138A (25)
Kananga in Kasai-Occidental	1/4 (25%)	3-F6	C		M184V (115)	A98G, V179D, Y181C (295)
Bukavu in Sud-Kivu	2/6 (33.3%)	3-I7	A		M184V (115)	K103N (120)
		3-I8	CRF25_cpx			G190A (130)
Mbandaka in Equateur	1/3 (33.3%)	4-G5	G			V179E (40)

Geographical analysis of DRM distributions suggested that the eastern part of DRC, specifically Goma had a higher prevalence of DRMs compared to Mbuji-Mayi, Lubumbashi, and Kinshasa (Table 2). These potential differences in the prevalence of DRM between the different regions was strictly a trend as no significant differences were detected due to sample size variations in these four regions. No DRMs that reduce susceptibility to drugs were observed in Kindu, Kisangani, and Matadi; however, the sample sizes obtained from these regions were limited. Further investigation with larger sample sizes is needed to verify potential differentials in the prevalence of DRMs in association with location.

## Discussion

To our knowledge, this study is the first to focus on characterizing HIV-1 genotypic diversity and the extent of circulating DRM among FSW in geographically distinct provinces of the DRC. Given the increasingly complex HIV recombinant forms reported to circulate in the DRC general population[6, 7], and the emergence of DRM coupled with virologic failures (VF)[15, 16], it is important to define contributory factors, and the potential role of high risk groups like FSW. Perhaps not surprising, we show that FSW have a higher HIV-1 prevalence than that reported for the general DRC population. They also harbor a higher prevalence of HIV-1 recombinants and DRM, and thus may be a significant source for development or spread of both back into the general population, in addition to their personal risk for exacerbated HIV pathogenesis.

Based on *env* sequencing, the predominant HIV-1 subtype found in FSW was A1, which is consistent with recent reports from the DRC general population[1, 8, 18]. We detected HIV-1 recombinants (35%), primarily in *pol* sequences, from DRC FSWs. This rate was comparable to that reported in a 2002 study[7]. Those CRFs were similar to genotypes previously reported in the general DRC population or from neighboring countries[7, 29]^,^ [30–35]. However, URF (CRF02/A/CRF02) was frequently detected in FSWs from Mbuji-Mayi/Lubumbashi in the South-central region of DRC. This suggests that specific CRF may be regionally circulating in FSWs and their clients. We also observed a high rate of discordance of subtype assignment (54%) in FSWs based on differential assignment of paired *pol* and *env* sequences. The discordance was similar in magnitude to that in a 2001 report (59.3%) from 27 infected non-FSWs from the Likasi province bordering Zambia[6], but greater than that reported in 2002 from the general population in the cities of Kinshasa, Lubumbashi, Kisangani, and Mbuyi-Mayi (41.1%)[7]. These data again suggest that FSW in DRC may be occupationally exposed to transmission of HIV-1 recombinants or may, in fact, be the source of those recombinants as a result of co-infection with diverse HIV-1 genotypes.

In addition, a higher prevalence of non-recombinant HIV-1 subtypes A1 and C was detected in Goma. The prevalence is similar to the predominant strains circulating in the bordering states of Rwanda and Burundi[36] [37] and could reflect genotypes introduced due to cross-border incursions as result of near constant conflict in the region. Similarly, subtype C predominates in Zambia, and we detected a trend toward increased prevalence of HIV-1 subtype C in the eastern and southern parts of DRC- the regions that border Zambia. These data suggest that factors affecting movement of infected people between neighboring countries, including trade, war and conflict, alter the regional spread of HIV-1 genotypes.

Perhaps most importantly, we identified resistance-associated mutations to all ART regimens commonly used in the region. These DRM were detected in 21.5% of the DRC FSWs sampled. Among those positive individuals, four had high-level resistance mutations to both NRTI and NNRTI. These mutations were also frequently observed among VF patients in the general population from the same cities[15]^,^ [16]. Moreover, we also observed the K65R mutation, which can compromise the efficacy of d4T in first-line regimens and ABC and ddI in second-line regimens in the DRC. Although no statistical significant differences were found in DRM regional distribution, Goma in Nord Kivu showed a trend toward higher prevalence of DRM when compared to other cities with high HIV prevalence. The prevalence of DRM observed in Goma was higher than that previously reported in ART-treated patients in Nord Kivu[16]. Although we could not determine whether mutations were transmitted or acquired due to the lack of treatment information, it is still important to determine the circulating, and therefore transmittable, DRM among sex workers who might develop or disseminate drug-resistant viruses.

Unfortunately, the study was limited by several caveats. Due to lack of clinical information associated with specimen collection from the study population, we were unable to determine whether DRM resulted from transmission of resistant variants or arose during infection and treatment in the FSW. In addition, because of the inherent difficulties in accessing FSW, coupled with limitations in laboratory capacity, cryogenic storage, and reliable transport infrastructure, DBS were the only reliable method for collection and preservation of samples for genotypic analysis. While the implementation of DBS prevented determination of HIV-1 plasma viral load and CD4 count, it provided the only workable method to gain unique preliminary insights into HIV-1 genotypic diversity in FSW from this resource-limited and war-torn country. Finally, the number of DBS collected was low in some cities. When combined with the lack of FSW participation and low prevalence of HIV-1 infection in those sampled, the result was under-sampling from some localities, thereby precluding rigorous statistical comparisons.

In conclusion, insights into the local development of HIV-1 genotypes and their transmission could be attained by studying high risk groups such as FSW. Nevertheless, our data suggest that HIV-1 genotypes in DRC FSW typically mirror the circulating virus in the general population. Alternately, the data may suggest the potential for FSW to be a source for development and spread of HIV-1 recombinants into the general population. Discriminating between these possibilities will require further investigation with a different sampling methodology. In addition, the geographic distribution of HIV-1 variants, the high prevalence of DRM in the eastern region and subtype C predominance in the southern region, all imply that cross-border HIV-1 introductions could modulate regional HIV-1 population dynamics via patronage of FSW. Given the complexity of the population structure of HIV-1 in DRC FSW coupled with apparent burden of resistance mutations, prophylaxis, and monitoring for recombination and drug resistance in this population will be necessary to limit development and spread of HIV-1 drug-resistance in DRC.

## Supporting information

S1 Table Number of HIV-1 sequences analyzed using dried blood spots collected from 10 cities in the DRC. (PDF)
