# Supplementary material for: HIV-1 subtypes and drug resistance mutations among female sex workers varied in different cities and regions of the Democratic Republic of Congo
Source: PLoS One. 2020 Feb 11;15(2):e0228670. doi: 10.1371/journal.pone.0228670 (PMC7012409; doi:10.1371/journal.pone.0228670)
Supplement: S1 Table — (PDF) [file pone.0228670.s001.pdf]

S1 Table. Number of HIV-1 sequences analyzed using Dried Blood Spots collected from 10 cities in the DRC

Cities	*Pol*	*Env*
Mbuji-Mayi	23	37
Lubumbashi	10	18
Kinshasa	13	22
Goma	25	32
Kananga	4	8
Kindu	2	5
Kisangani	4	5
Bukavu	6	9
Mbandaka	3	5
Matadi	3	4
Total	**93**	**145**
